# Age-dependent association of γ-crystallins with aged α-crystallins from old bovine lens

**Published:** 2008-05-19

**Authors:** Larry Takemoto, Aldo Ponce, Christopher M. Sorensen

**Affiliations:** 1 Division of Biology, Kansas State University, Manhattan, KS; 2 Department of Physics, Kansas State University, Manhattan, KS

## Abstract

**Purpose:**

Previous theoretical and experimental studies have predicted that the loss of weak protein interactions between α- and γ-crystallins could result in a decrease in the transparent properties of the aging lens.

**Methods:**

α-Crystallins were prepared from the nucleus of old bovine lens, and γ-crystallins were prepared from whole fetal bovine lens or from the nucleus of old bovine lens. The possible interactions of old α-crystallins with either old γ-crystallins or fetal γ-crystallins were quantitated at equilibrium using microequilibrium dialysis. The amount of each γ-crystallin species in the “full” versus “empty” chambers was determined by reverse phase chromatography to obtain a binding ratio (full/empty).

**Results:**

A binding ratio greater than 1.00 is indicative of a α-crystallin/γ-crystallin interaction. Within experimental error (±2X standard deviation), there were no interactions between aged γ-crystallins with aged α-crystallins while there were significant interactions between some of the fetal γ-crystallins with aged α-crystallins.

**Conclusions:**

In the aged bovine lens, when transparency is known to decrease, there is no detectable interaction of γ-crystallins with α-crystallins as measured by microequilibrium dialysis. The results are consistent with the hypothesis that short-range, weak, attractive interactions between α- and γ-crystallins are necessary for maximum transparency of the lens.

## Introduction

The visual acuity of the eye is dependent upon the transparent and refractile properties of the lens. Although the ocular lens comprises very high concentrations of protein (i.e., greater than 300 mg/ml), this tissue is highly transparent in young organisms. As the organism ages, the lens gradually loses some transparency [1]. In many humans older than 60 to 65 years of age, loss of transparency is sufficient to result in cataractogenesis.

The biophysical/biochemical basis for lens transparency in the young lens is a necessary precondition to understanding on a molecular basis why the lens loses some of its transparency during normal aging and why much of its transparency is lost during cataractogenesis. X-ray scattering of increasing concentrations of lens proteins showed the expected increase scattering up to approximately 120 mg/ml, as the protein concentration exceeded 120 mg/ml and approached that existing in the intact lens, the scattering decreased [2]. Theoretical calculations by Bettelheim et al. [3], which assumed a model system of spheres, came to a similar conclusion. Both studies hypothesized that the concentration-dependent decrease in scattering at high protein concentrations was due to the liquid-like, short-range order of protein molecules and the concomitant lack of large scale fluctuations.

How this short-range order might be perturbed during aging and cataractogenesis was addressed by Ponce et al. [4]. They suggested that heterologous, protein–protein, attractive interactions between lens crystallins such as α-crystallins and γ-crystallins could result in uniform protein density across the lens to ensure minimal scattering of light. Studies using a two-hybrid assay system [5,6], filtration [7], light scattering [8], and microequilibrium dialysis [9] have indeed demonstrated the existence of these weak attractions. Furthermore, light scattering studies have demonstrated that at high concentrations, mixtures of α- and γΒ-crystallin scatter less light than the individual component solutions [10]. On the other hand, during aging and/or cataractogenesis, increased heterologous, attractive interactions between subpopulations of lens crystallins could result in large protein density fluctuations likely through cluster formation with resulting scattering of light.

Recently, Stradner et al. [11] have used neutron scattering combined with molecular dynamics simulations to confirm that weak, attractive protein interactions between α- and γ-crystallins are indeed necessary for the transparent properties of the lens. These studies suggested that heterologous interactions resulting in lens transparency were in a relatively narrow window of binding energy. Abnormally strong interactions could result in the formation of high molecular weight aggregates and scattering of light as has been hypothesized by Benedek [12]. Such a condition could be especially applicable to lens cataractogenesis [13].

Alternatively, very weak interactions or no interactions at all could also result in protein density fluctuations due to the well documented interaction of γ-crystallins with themselves [14,15] and the depletion interaction of α-crystallins [11]. The result would also be a decrease in lens transparency.

To test whether a loss of heterologous, crystallin interactions could be correlated with loss of transparency, we have used microequilibrium dialysis to measure possible interactions of α- and γ-crystallins from the nucleus of the aged bovine lens, which is not totally opaque but does show decreases in lens transparency relative to the fetal lens. The results show no detectable interactions of aged γ-crystallins with aged α-crystallins, consistent with the prediction that weak heterologous interactions of α- and γ-crystallins are necessary for maximum transparency in the lens.

## Methods

Lenses from fetal calves and from aged cows approximately 30 months of age were obtained from Antech Inc. (Tyler, TX). The lenses were stored at −80 °C until use. As previously described [16], α- and γ-crystallins were prepared from the water-soluble fraction from whole fetal lenses and from the nucleus of aged lenses using a TSK G3000SW gel filtration column (Tosoh Bioscience, Montgomeryville, PA). Resulting crystallin preparations were concentrated using Aquacide I (Calbiochem, LaJolla, CA) then dialyzed extensively against microequilibrium dialysis buffer (10 mM Tris hydrochloride, 0.1 mM dithiothreitol, 3 mM sodium azide, and 0.15 M sodium chloride, pH 7.4). Protein concentration was determined according to Bradford [17] using BSA as standard.

Microequilibrium dialysis used the dialysis cell from Nest Group (Southborough, MA). The cell contained two reservoirs of 50 μl capacities, separated by a regenerated cellulose membrane of 100 kDa cut-off (Millipore, Bedford, MA). α-Crystallin (4.0 mg/ml) and γ-crystallin (2.0 mg/ml) in microequilibrium dialysis buffer were placed in one cell, and buffer alone was placed in the other cell. The dialysis cell was incubated for five days at 37 °C to allow for complete equilibration of γ-crystallins across the membrane as previously described [9].

At the end of five days of incubation, the contents of each cell were carefully removed and 5 μl were analyzed by reverse phase HPLC using a C_18_ reverse phase column (Vydac, 4.6×250 mm, 300 Angstrom pore size; Phenomenex, Torrance, CA) and a linear gradient of 32%–37% v/v acetonitrile in water all of which contained 0.1% v/v trifluoroacetic acid, over a period of 40 min. The area of each peak was used to compute a binding ratio (full/empty) as previously described [9].

## Results

**Figure 1:**
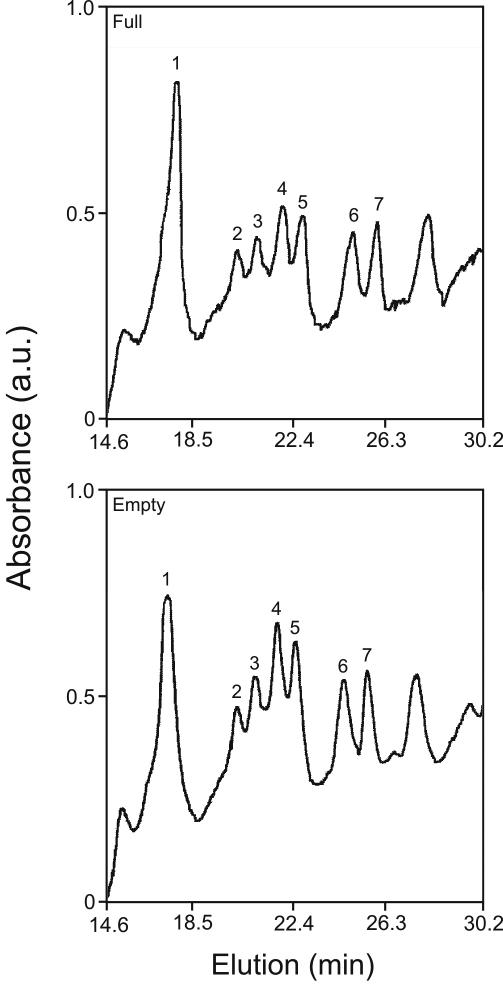
Old γ-crystallins binding to old α-crystallins. Elution profiles are shown of γ-crystallins in the full (top) versus empty (bottom) chambers of microequilibrium dialysis of old α-crystallins and old γ-crystallins. See Methods for details of C_18_ reverse phase chromatography to resolve γ-crystallin peaks. Numbered peaks were quantitated and used to compute binding ratios listed in Table 1.

Figure 1 shows an elution profile of the γ-crystallin fraction from the nucleus of old lens that was incubated with α-crystallins from the nucleus of old lens. Samples were taken either from the chamber containing α-crystallin (full chamber, top) or from the chamber not containing α-crystallin (empty chamber, bottom). Reverse phase chromatography resolved the γ-crystallin fraction into at least seven peaks (numbered 1–7). Since α-crystallins cannot cross the membrane between the two chambers and if there is any interaction of α-crystallins with any of the γ-crystallins, the amount of γ-crystallin in the chamber containing α-crystallins (i.e., full chamber) should be greater than the corresponding amount in the chamber not containing α-crystallins (i.e., empty chamber).

Table 1 shows the average of triplicate determinations for each peak (±SD). A full/empty ratio of 1.00 indicates no interactions between the specific γ-crystallin species and α-crystallins. Assuming a parametric distribution, (±2X SD) should include 95.4% of all full/empty ratios [18]. Based upon the results of Table 1, all of the full/empty ratios would overlap this number (regular type), suggesting that there are no interactions between any of the γ-crystallins from old lens and α-crystallins from old lens.

**Table 1:** Binding Ratios (full/empty) of old α-crystallins to old γ-crystallins, and binding of old α-crystallins to fetal calf γ-crystallins.

**Peak Number**	**Full/Empty (old α-crystallin, old γ-crystallin)**	**Full/Empty (old α-crystallin, fetal calf γ-crystallin)**
1	1.19 ± 0.12	**1.54 ± 0.15**
2	1.48 ± 0.58	**1.61± 0.27**
3	1.13 ± 0.19	0.86 ± 0.097
4	1.24 ± 0.15	**1.23 ± 0.06**
5	1.14± 0.072	**1.32 ± 0.083**
6	0.93 ± 0.069	1.7 ± 0.48
7	0.87 ± 0.083	1.45 ± 0.11

Full/empty ratios in regular type represent ratios that are not statistically significant, compared with a ratio of 1.0, while full/empty ratios in **bold** type represent ratios that are statistically significant, compared with a ratio of 1.0. See text for a further explanation.

**Figure 2:**
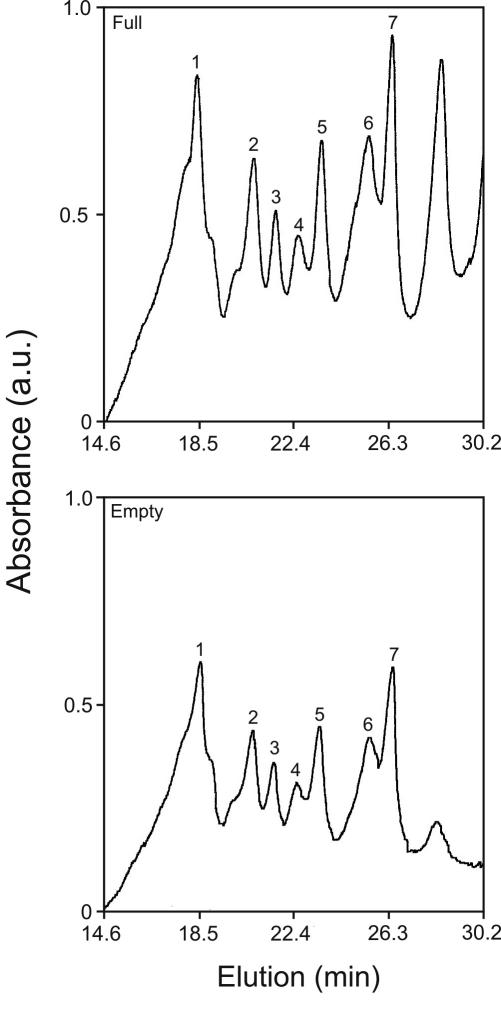
Fetal γ-crystallin binding to old α-crystallins. Elution profiles of γ-crystallins in the full (top) versus empty (bottom) chambers of microequilibrium dialysis of old α-crystallins and fetal calf γ-crystallins. See Methods for details of C_18_ reverse phase chromatography to resolve γ-crystallin peaks. Numbered peaks were quantitated and used to compute binding ratios listed in Table 1.

For comparison, Figure 2 and Table 1 also include the full/empty ratios for microequilibrium dialysis analysis of fetal γ-crystallins incubated with old α-crystallins. The ratios for peaks 1, 2, 4, 5, and 7 are all greater than 1.00 when including ratios within the range (±2X SD; bold type in Table 1). The number of γ-crystallin species showing significant interactions with α-crystallins is similar to previous microequilibrium dialysis studies of fetal bovine γ-crystallins incubated with fetal bovine α-crystallins [16].

## Discussion

Taken together, the results of Figure 1, Figure 2, and Table 1 demonstrate that none of the γ-crystallin species from old bovine lens interacts with α-crystallins from old lens while the same analytical techniques show significant interactions between many of the gamma species from fetal bovine lens when incubated with α-crystallins from old lens. Previous studies have shown that determination of protein amount in the full versus empty chambers can identify significant interactions of two fetal lens proteins at equilibrium [9]. Within experimental error, this conclusion is independent of the amount of protein since the ratio of the protein amount in both the full and empty chambers during equilibrium acts as an internal control [9]. We therefore conclude that an attractive interaction between alpha and γ-crystallins is necessary for lens transparency.

This conclusion is consistent with a recent study by Stradner et al. [11] who used neutron scattering and molecular dynamic simulations to characterize the reasons for lens transparency. Modeling using molecular dynamic simulations was able to reproduce experimental neutron scattering results when there existed a weak, attractive interaction between γ- and α-crystallins. Abnormally low interactions or abnormally strong interactions resulted in increased scattering and regions of protein density fluctuation.

Although lack of α-crystallin/γ-crystallin interactions could result in density fluctuations and a decrease in transparency, it is possible that abnormally strong attractive interactions of crystallins could result in the formation of high molecular weight aggregates that have been found in both aging normal lenses and cataractous lenses [19]. These regions of higher protein density could scatter light because of their higher refractive index and/or directly scatter light because of their large size as hypothesized by Benedek [12]. Implicit in this model is the assumption that an abnormally strong interaction between crystallins is not uniform across the lens but rather is localized to different regions of the lens to differing degrees. Biochemical analysis of aged and cataractous lens proteins has indeed found that strong interactions of lens proteins result in the formation of aggregates of varying size [20]. Similarly, Stradner et al. [11] describe the nonmonotonic functionality of transparency with alpha-gamma interaction such that no interaction leads to turbidity, weak interaction leads to transparency, and too strong interaction leads once again to turbidity. This last situation could cause aggregation.

The biochemical reasons for the age-related change in the interactions of α-crystallins with γ-crystallins seen in the present study probably involve posttranslational modifications occurring during the aging process. Previous studies have demonstrated covalent changes occurring at both the NH_2_-terminus [21] and COOH-terminus [22-25] of αΑ-crystallin during the aging of bovine lens. A quantitative correlation of these and possibly other covalent changes in the α-crystallin molecule with changes in its interaction with γ-crystallins should lead to a better understanding of the molecular mechanisms involved in transparency loss in the aging lens.

## References

[1] Bron AJ, Vrensen GF, Koretz J, Maraini G, Harding JJ (2000). The ageing lens.. Ophthalmologica.

[2] Delaye M, Tardieu A (1983). Short-range order of crystallin proteins accounts for eye lens transparency.. Nature.

[3] Bettelheim FA, Siew EL (1983). Effect of change in concentration upon lens turbidity as predicted by the random fluctuation theory.. Biophys J.

[4] Ponce A, Sorensen C, Takemoto L (2006). Role of Short-Range Protein Interactions in Lens Opacifications. Mol Vis.

[5] Fu L, Liang JJ (2002). Detection of protein-protein interactions among lens crystallins in a mammalian two-hybrid system assay.. J Biol Chem.

[6] Fu L, Liang JJ (2003). Alteration of protein-protein interactions of congenital cataract crystallin mutants.. Invest Ophthalmol Vis Sci.

[7] Biswas A, Das KP (2004). Role of ATP on the interaction of alpha-crystallin with its substrates and its implications for the molecular chaperone function.. J Biol Chem.

[8] Bettelheim FA, Chen A (1998). Thermodynamic stability of bovine alpha-crystallin in its interactions with other bovine crystallins.. Int J Biol Macromol.

[9] Ponce A, Takemoto L (2005). Screening of crystallin-crystallin interactions using microequilibrium dialysis.. Mol Vis.

[10] Thurston GM (2006). Liquid-liquid phase separation and static light scattering of concentrated ternary mixtures of bovine alpha and gammaB crystallins.. J Chem Phys.

[11] Stradner A, Foffi G, Dorsaz N, Thurston G, Schurtenberger P (2007). New insight into cataract formation: enhance stability through mutual attraction.. Phys Rev Lett.

[12] Benedek GB (1971). Theory of transparency of the eye.. Appl Opt.

[13] Peterson J, Radke G, Takemoto L (2005). Interaction of lens alpha and gamma crystallins during aging of the bovine lens.. Exp Eye Res.

[14] Tardieu A, Veretout F, Krop B, Slingsby C (1992). Protein interactions in the calf eye lens: interactions between beta-crystallins are repulsive whereas in gamma-crystallins they are attractive.. Eur Biophys J.

[15] Stevens A, Wang SX, Caines GH, Schleich T (1995). ^13^C-NMR off-resonance rotating frame spin-lattice relaxation studies of bovine lens gamma-crystallin self association: effect of macromolecular crowding.. Biochim Biophys Acta.

[16] Takemoto LJ, Ponce AA (2006). Decreased association of aged alpha crystallins with gamma crystallins.. Exp Eye Res.

[17] Bradford MM (1976). A rapid and sensitive method for the quantitation of microgram quantities of protein utilizing the principle of protein-dye binding.. Anal Biochem.

[18] Montgomery R, Swenson CA. Dimensional analysis and experimental measurements. In: Montgomery R, Swenson CA, editors. Quantitative problems in the biochemical sciences. San Francisco: W.H. Freeman and Company; 1969. p. 46–47.

[19] Jedziniak JA, Kinoshita JH, Yates EM, Hocker LO, Benedek GB (1973). On the presence and mechanism of formation of heavy molecular weight aggregates in human normal and cataractous lenses.. Exp Eye Res.

[20] Takemoto LJ, Azari P (1977). Isolation and characterization of high molecular weight proteins from human cataractous lens.. Exp Eye Res.

[21] Takemoto L, Horwitz J, Emmons T (1992). Oxidation of the N-terminal methionine of lens alpha-A crystallin.. Curr Eye Res.

[22] Emmons T, Takemoto L (1992). Age-dependent loss of the C-terminal amino acid from alpha crystallin.. Exp Eye Res.

[23] Takemoto L, Gopalakrishnan S (1994). Alpha-A crystallin: Quantitation of C-terminal modification during lens aging.. Curr Eye Res.

[24] Takemoto LJ (1995). Identification of the in vivo truncation sites at the C-terminal region of alpha-crystallin from aged bovine and human lens.. Curr Eye Res.

[25] Takemoto L (1999). Increased cleavage of the C-terminal serine from alpha-A crystallin present in the high molecular weight aggregate fraction from human and bovine lenses.. Curr Eye Res.

